# Distinct response to IL-1β blockade in liver- and lung-specific metastasis mouse models of pancreatic cancer with heterogeneous tumor microenvironments

**DOI:** 10.1186/s40164-025-00607-w

**Published:** 2025-02-13

**Authors:** Sophia Y. Chen, Heng-Chung Kung, Birginia Espinoza, India Washington, Kai Chen, Kaiyi Mu, Haley Zlomke, Michael Loycano, Rulin Wang, William R. Burns, Juan Fu, Lei Zheng

**Affiliations:** 1 Department of Oncology and The Sidney Kimmel Comprehensive Cancer Center, The Johns Hopkins University School of Medicine, Baltimore, MD USA; 2 Pancreatic Cancer Precision Medicine Center of Excellence Program, The Johns Hopkins University School of Medicine, Baltimore, MD USA; 3 Department of Surgery, The Johns Hopkins University School of Medicine, Baltimore, MD USA; 4 Mays Cancer Center, University of Texas, 7979 Wurzbach Road, MC8026, San Antonio, TX 78229 USA

## Abstract

**Background:**

Pancreatic ductal adenocarcinoma (PDAC) is characterized by a heterogeneous tumor microenvironment (TME). The mechanism by which this heterogeneity confers resistance against immunotherapy remains unclear. Interleukin- 1β (IL-1β) is a proinflammatory cytokine that regulates heterogeneous cancer associated fibroblast (CAF) phenotype and promotes an immunosuppressive TME. Anti-IL-1β monoclonal antibody significantly enhanced the anti-tumor activity of anti-PD-1 in a preclinical model of PDAC. However, clinical trials have shown limited activity of the anti-IL-1β and anti-PD-1 combination. Therefore, we hypothesize that anti-tumor immune response to the combination of anti-IL-1β and anti-PD-1 antibodies is context-dependent and would be affected by the TME heterogeneity in PDAC.

**Methods:**

Liver- and lung-specific metastasis mouse models of PDAC were used to investigate the antitumor activity of anti-IL-1β and anti-PD-1 antibodies alone or in combination by ultrasound examination and survival analysis. Their effects on the TME heterogeneity were assessed by flow cytometry and single nuclear RNA sequencing.

**Results:**

The combination of anti-IL-1β and anti-PD-1 antibodies does not slow primary tumor growth but prolongs overall survival and reduces lung metastasis rates in a PDAC orthotopic murine model with lung metastasis tropism. In contrast, combination therapy slows primary tumor growth and prolongs survival, but does not reduce liver metastasis rates in a PDAC murine orthotopic model with liver metastasis tropism. Flow cytometry analysis showed that the combination of anti-IL-1β and anti-PD-1 antibodies restores T cell activation negated by the monotherapies. Mechanistically, in the PDAC model with lung metastasis tropism, but not in the model with liver metastasis tropism, combination treatment reverses an increased trend of immunosuppressive myeloid cells as a result of monotherapy. Single-nuclear RNA sequencing analysis of both organ-specific tumor models demonstrated that anti-IL-1β treatment altered infiltration and function of CAF and immune cells differently. Furthermore, anti-IL-1β treatment modulated cytokine/chemokine ligand-receptor-receptor interactions in the models with different organ-specific metastasis distinctly.

**Conclusion:**

This study reveals the differential responses of organ-specific metastasis mouse models of PDAC with distinct TMEs to anti-IL-1β and anti-PD-1 treatments, suggesting that treatment response is context-dependent and affected by TME heterogeneity.

**Supplementary Information:**

The online version contains supplementary material available at 10.1186/s40164-025-00607-w.

## Background

Pancreatic ductal adenocarcinoma (PDAC) is characterized by dynamic and pronounced changes in the tumor microenvironment (TME) as a result of tumor progression [1]. Though components of the PDAC TME, including tumor-infiltrating immune cells, cancer associated fibroblasts, vasculature, and extracellular matrix (ECM), are observed in other solid tumors, each of these components is known to play critical roles in PDAC growth, metastases, and response to therapy [2].

Due to the desmoplastic stroma, poor T cell infiltration, and immunosuppressive TME, immune checkpoint inhibitors such as anti-PD-1/PD-L1 have failed to demonstrate effective or promising clinical outcome in PDAC compared to other malignancies [3, 4]. Of particular importance, though heterogeneity within the stroma of PDAC has been well established, its contributions to the primary resistance of PDAC to immunotherapy remains relatively unknown [5–7]. Given these challenges, there is an urgent need to identify agents that can successfully target the PDAC stroma, overcome the immunosuppressive TME, and synergize with available immunotherapies.

Stromal heterogeneity in PDAC was first characterized by the identification of three CAF phenotypes in the TME, including inflammatory CAF (iCAF), myofibroblastic CAF (myCAF) and antigen presenting CAF (apCAF) [8–10]. myCAFs are located adjacent to the neoplastic cells, express high levels of alpha smooth muscle actin, and secrete factors such as collagen and hyaluronan that make up the ECM [8, 11]. On the other hand, iCAFs are located more distantly from neoplastic cells and secrete inflammatory factors such as interleukin (IL-) 6 [10]. More recently, single-cell analyses have identified other subpopulations of CAFs with different characteristics and association prognosis [12–14]. Most notably, apCAFs were found to express major histocompatibility complex (MHC) II and CD74 and may induce CD4 + T cell differentiation into regulatory T cells (Tregs) [9, 15]. While myCAF differentiation is driven by transforming growth factor β (TGFβ) signaling, tumor-secreted IL-1 induces LIF expression and activates downstream JAK/STAT signaling to generate iCAFs [10]. iCAFs continue to promote inflammation through secreted cytokines and have also been shown to drive M2 macrophage polarization and attract immunosuppressive cells such as myeloid-derived suppressor cells (MDSCs) to the TME [16–18]. Thus, disrupting the pro-tumor properties of iCAFs remain a topic of interest with IL-1 as a major target.

IL-1 is a proinflammatory cytokine and plays a critical role in regulating inflammatory responses [19]. However, high levels of IL-1 can result in chronic inflammation, which has been implicated in tumorigenesis, cancer progression, and metastasis [20, 21]. Particularly, IL-1β, the β subunit of IL-1, has been demonstrated to significantly alter the TME and subverting anti-tumor immunity beyond directly promoting tumor cell proliferation [20, 22, 23]. In addition to regulating the generation of iCAFs, IL-1β-driven inflammation has been shown to induce an immunosuppressive TME by increasing the infiltration of immunosuppressive cells such as MDSCs and Tregs [24]. A prior study also found that IL-1β was essential in promoting desmoplasia and establishing the pro-tumorigenic TME mediated by M2 macrophages, regulatory B cells, and Th17 cells [25]. The study also identified that tumor cell expression of IL-1β is driven by microbial-dependent activation of toll-like receptor 4 (TLR4) signaling and subsequent engagement of the NLRP3 inflammasome [25]. Clinically, high intra-tumoral and serum IL-1β levels have been associated with increased chemoresistance and worse outcomes in PDAC [26–28].

Due to the immunosuppressive properties of IL-1β and the advent of immune checkpoint blockade, there has been growing interest in the combination of anti-IL-1β and anti-PD-1 therapies, which has been tested in various preclinical models. Particularly, Canakinumab, a fully human monoclonal antibody against IL-1β, in combination with Pembrolizumab anti-PD-1 antibody delayed tumor growth and increased infiltration of CD8 T cells in a humanized bone marrow liver thymic non-small cell lung cancer model [29]. Similarly, anti-IL-1β monoclonal antibody significantly enhanced the anti-tumor activity of anti-PD-1 antibodies in a preclinical model of PDAC, which was accompanied by increased infiltration of CD8 T cells into the tumor [25].

While preclinical studies have demonstrated the potential benefits of anti-IL-1β monoclonal antibody therapy, these results have not been necessarily translated to clinical benefits in patients. The recently completed phase III CANOPY-1 trial found that the addition of Canakinumab to first line Pembrolizumab did not result in increased progression free survival and overall survival for patients with non-small cell lung cancer [30]. A Phase Ib trial demonstrated the safety and feasibility of gemcitabine, nab-paclitaxel, Canakinumab, and human anti-PD-1 Spartalizumab combination therapy in patients with metastatic PDAC [31]. Analysis of peripheral blood and serum from treated patients only revealed a modest reactivation of peripheral CD8 T cells and reduced circulating monocytic MDSCs [32]. The combination of Canakinumab with standard of care chemotherapy and Tislelizumab (humanized anti-PD-1) is currently being tested in another Phase Ib trial as well (NCT05984602). Though these trials demonstrate that anti-IL-1β plus anti-PD-1 combination therapy is tolerable in patients, they have not provided compelling evidence supporting its efficacy in PDAC.

Therefore, we hypothesize that anti-tumor immune response to the combination of anti-IL-1β and anti-PD-1 antibodies is context-dependent and would be affected by the stromal heterogeneity in PDAC. Here, we report the efficacy of IL-1β inhibition in combination with anti-PD-1 therapy on orthotopic murine PDAC models with different metastatic tropism and the distinct effect of combination therapy on the TME of each model.

## Methods

### Cell lines

The KPC-4545 and KPC-3403 tumor cell lines are an established PDAC cell line derived from a KPC transgenic mouse model in a C57BL/6 background with tissue-specific Kras and p53 knock-in mutations as previously described [33]. The KPC-4545 tumor cell line is derived from primary tumors of a KPC mouse with liver metastases only, and the KPC-3403 tumor cell line is derived from primary tumors of a KPC mouse with lung metastases only, as previously described [34]. The KPC cell lines were cultured in RPMI 1640 media (Life Technologies) with 10% fetal bovine serum (FBS, Benchmark), 1% MEM-NEAA (minimum essential medium-non-essential amino acids, Life Technologies), 1% penicillin/streptomycin (Life Technologies), 1% sodium pyruvate (Sigma), and 1% L-glutamine (Life Technologies), maintained at 37 °C in 5% CO_2_. Harvested tumor-infiltrating immune cells were processed in T cell media consisting of RPMI 1640 (Life Technologies) with 10% FBS, 1% MEM-NEAA, Life Technologies), 1% penicillin/streptomycin (Life Technologies), 1% L-glutamine (Life Technologies), and 0.05% 2-mercaptoethanol (Sigma-Aldrich).

### Mice and in vivo experiments

All animal experimental procedures were approved by the local Institutional Animal Care and Use Committee (IACUC) and were conducted in accordance with the guidelines set by the Association for Assessment and Accreditation of Laboratory Animal Care. C57BL/6 mice (6–8 weeks old) mice were purchased from Jackson Laboratories. Mice were monitored at least once a day for survival data purposes. Mice with observed signs of distress including hunched posture, lethargy, dehydration, and rough hair coat were considered to have reached “survival endpoint” and consequently euthanized.

### Orthotopic murine model and treatment regimen

The KPC PDAC orthotopic model has been previously described. Briefly, 2 × 10^6^ KPC cells were injected subcutaneously in the flanks of syngeneic female C57Bl/6 mice. The subcutaneous tumors were harvested after 1–2 weeks and cut into 2–3 mm^3^ pieces. New 6–8 weeks old syngeneic female C57Bl/6 mice were anesthetized. A left subcostal incision was created to enter the abdomen and access the body and tail of the pancreas. Using micro scissors, one 2–3 mm^3^ piece of subcutaneous tumor was implanted within a small pocket in the distal body and tail portion of the pancreas and secured with 7 − 0 Prolene suture. The peritoneum and skin were sutured in two layers with 4 − 0 sutures.

Anti-IL-1β antibodies (Bristol Meyers Squibb) and isotype control anti-human IgG antibodies (Bristol Meyers Squibb) were administered intraperitoneally (IP) at 10 mg/kg per dose on days 7, 9, 11, 13, 15, 17, 19 and 21 after orthotopic tumor implantation. Anti-mouse PD-1 antibodies (Bristol Meyers Squibb) and anti-mouse mIgG1 isotype control antibodies (Bristol Meyers Squibb) were administered IP at 5 mg/kg per dose on days 7, 10, 14, 17, 21, and 24. Tumor size was measured by Vevo 3100 ultrasound in the transverse and longitudinal orientations with respect to the transducer.

### Cell processing and flow cytometry

Murine orthotopic pancreatic tumors were resected on day 13 after tumor implantation for analysis of tumor-infiltrating immune cells as previously described. Each tumor was mechanically and enzymatically processed using the mouse Miltenyi Tumor Dissociation Kit (Miltenyi Biotec) and gentleMACS Octo Dissociator (Miltenyi Biotec), filtered through a 70 mm strainer, and brought to a volume of 20 mL in T cell medium. Cell suspensions were centrifuged at 1500 rpm for 5 min. Cell pellets were suspended in 4 mL of Ammonium-Chloride-Potassium lysis buffer (Quality Biological) and spun at 1500 rpm for 5 min. Cell pellets were then resuspended in 6 mL of 80% Percoll (Cytiva), overlaid with 6 mL of 40% Percoll, and centrifuged for 25 min at 3200 rpm without break at room temperature. The leukocyte layer was removed and quenched with T cell media.

Following isolation of tumor-infiltrating immune cells from murine pancreatic tumor, cells were stained with Live/Dead Aqua (Invitrogen) for 30 min on ice, washed twice with PBS, and then blocked with anti-mouse Fc antibody (BD Biosciences) in FACS buffer for 10 min on ice. The cell surface antibodies used were: CD45-PerCP Cy5.5 (BioLegend), CD8-PE Cy7 (BioLegend), CD4-APC Fire (BioLegend), CD25-BV421 (BioLegend), CD45-FITC (BioLegend), CD3-APC Cy7 (BioLegend), CD4-BV650 (BioLegend), CD11b-PE Texas Red (Thermo Fisher), Ly6C-PerCP Cy5.5 (BioLegend), Ly6G-V450 (BD Biosciences), F4/80-PE Cy7 (BioLegend), OX40-FITC (BioLegend), LAG3-PE (BioLegend), TIM3-PE CF594 (BD Biosciences), CD137-APC (BioLegend), and PD-1-BV421 (BioLegend).

Intracellular staining for forkhead box P3 (FoxP3) was performed following cell surface marker incubation. Cells were suspended in cold Fixation/Permeabilization solution (eBioscience) and incubated for 30 min on ice at 4 °C. Cells were then washed twice with Permeabilization/Wash buffer (eBioscience). FoxP3-PE antibody (BioLegend) was added, and the cells were incubated for 40 min on ice. Cells were then washed twice in Permeabilization/Wash buffer and resuspended in FACS buffer. All flow cytometry experiments were performed using CytoFLEX (Beckman Coulter), and flow data was analyzed using CytExpert software (Beckman Coulter).

### Nuclei isolation for single nuclear RNA sequencing (snRNA-seq)

Single nuclei were isolated from KPC PDAC tumors based on a modified 10X Genomics protocol previously described in our study [35]. In brief, sucrose density buffer consisting of sucrose (0.344 g/mL, Sgima-Aldrich), 10 mM HEPES (Gibco), 5 mM CaCl_2_ (Sigma-Aldrich), 3 mM magnesium acetate tetrahydrate (Sigma-Aldrich), 1 mM DTT (Sigma-Aldrich), and 0.2 U/mL NxGen RNase inhibitor (Lucigen) was prepared fresh prior to single-nuclei isolation. Frozen KPC tumors were homogenized into powder using mortar and pestle in liquid nitrogen and lysed with buffer containing containing 0.1% Triton-X (Sigma-Aldrich), 0.2 U/mL NxGen RNase inhibitor (Lucigen), and sucrose density buffer on ice for 3 min. The crude homogenate was then passed through a 100 μm cell strainer, washed with sucrose density buffer, and centrifuged at 400 g for 5 min at 4 °C (low brake). The supernatant was aspirated, and the pellet was resuspended in PBS + 1% BSA (Sigma-Aldrich), and 0.2 U/µL RNase inhibitor (Lucigen) and filtered through a 35 μm flow filter tube. Chromium Next GEM Single Cell V(D)J Reagent Kits v.1.1 was used for cDNA amplification per the manufacturer’s instructions.

### snRNA-seq analysis

snRNA-seq data was processed and analyzed as previously described in our study [35]. Briefly, snRNA-seq data from two distinct batches (KPC-3403 and KPC-4545) were generated, with each batch consisting of tumors from six treatment groups BCL files were converted to FASTQ format with Illumina’s bcl2fastq, and FASTQ reads were demultiplexed and aligned to the GRCm39-mm10 transcriptome with the “include-intron” option to account for intronic reads and maximize sensitivity with CellRanger v6.1.2. Unique molecular identifiers (UMIs) and nuclei barcodes were extracted to output a digital gene expression (DGE) matrix for each individual sample. Data from 4 treatment groups including the control, anti-IL-1β, anti-PD-1, and anti-IL-1β/anti-PD-1 combo treatment groups were isolated out for analysis in this study. CellRanger output showed fraction reads in cells (fraction of read with valid barcodes confidently mapped and associated with a cell) between 15.3 and 47.1% and fraction reads confidently mapped to transcriptome ranging from 4.7 to 32.4% across 4 treatment groups including the control, anti-IL-1β, anti-PD-1, and anti-IL-1β + anti-PD-1 combo treatment groups in both KPC-3403 and KPC-4545 models. Identification of highly variable genes, dimensional reduction, unsupervised clustering, and downstream analyses were performed using Seurat v4.1.0.

Ambient RNA and other technical artifacts were removed for each individual sample with CellBender remove-background on Terra workspace. Nuclei from all of six samples of each batch were merged with the Seurat merge function, and nuclei with less than 200 features or more than 2500 features were removed. Gm42418 and AY036618 were excluded from subsequent analysis due to their overlap with rRNA element Rn45s, representing possible rRNA contamination. Counts were log-normalized with a scale factor of 10,000, and the 2000 most variable genes were identified and used for principal component analysis. Nuclei were clustered at a resolution of 0.8 and visualized through uniform manifold approximation and projection (UMAP). No significant batch effects between samples were observed. Distinct clusters identified were annotated using known cell-type-specific gene markers curated from literature. The four anti-IL-1β study samples were then extracted for downstream comparisons for each of the two models.

Differential gene expression between the same cell types of different treatment samples was performed with the FindMarkers function and MAST test from Seurat. Significantly differentiated genes (log_2_FC > 0.5 or log_2_FC < -0.5 and adjusted p-value < 0.05) were used for functional pathway analysis with Metascape. A threshold of false-discovery-rate (FDR) adjusted p-value < 0.05 was used to determine significant pathways. iTALK v0.1.0 was used to infer ligand-receptor interactions between CAFs, CD8 T cells, macrophage, and neutrophils with human homologs for each individual sample. The top 30 ligand receptor pairs for each category defined by iTALK (growth factor, cytokines, checkpoints, and others) were visualized and compared between different samples.

## Results

### The combination of anti-IL-1β and anti-PD-1 treatment does not slow primary tumor growth but prolongs overall survival in PDAC orthotopic murine model with metastatic lung tropism

**Fig. 1:**
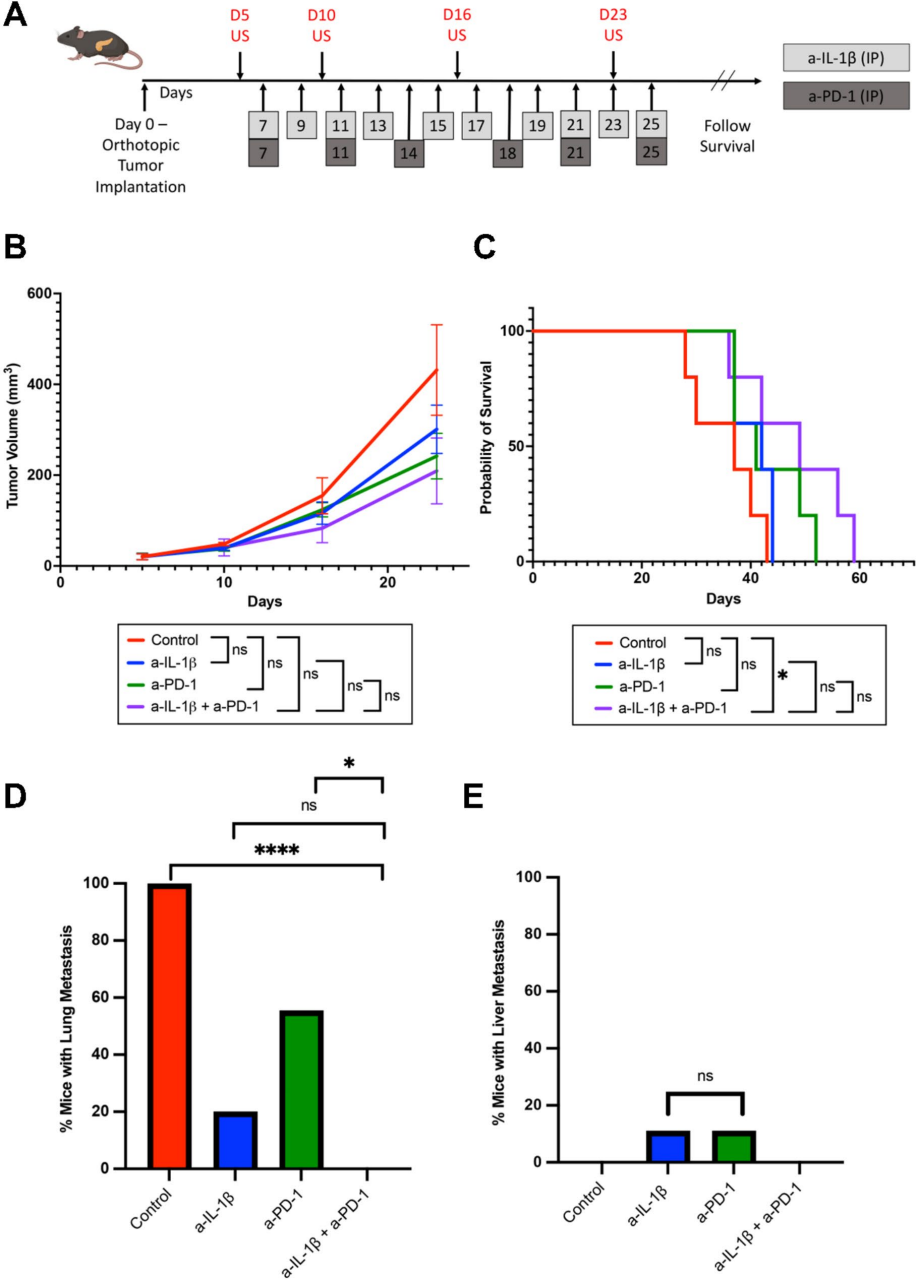
The combination of anti-IL-1β and anti-PD-1 antibodies prolonged survival and reduced lung metastasis rate in orthotopic PDAC mouse model with lung metastasis potential. (**A**) Treatment schema for PDAC orthotopic model tumor growth experiment using anti-IL-1β. Pre-treatment US was performed on day 5. Mice were treated with anti-IL-1β (10 mg/kg i.p. twice/week), anti-PD-1 (5 mg/kg i.p. twice/week), or IgG control (5 mg/kg i.p. twice/week) for three weeks. (**B**) PDAC orthotopic tumor size was evaluated by US weekly until day 29 in mice treated with different combinations of anti-IL-1β and anti-PD-1 (*n* = 5 mice per group). (**C**) Kaplan-Meier survival curves of PDAC orthotopic model mice treated with different combinations of anti-IL-1β and anti-PD-1 (*n* = 5 mice per group). (**D**&**E**) When PDAC orthotopic tumor mice reached survival endpoint, necropsies were performed, and the number of mice with lung metastases (**D**) and liver metastases (**E**) for the KPC-3403 lung metastasis potential model were identified grossly and histologically (*n* = 8–10 mice per group). US, ultrasound. Unpaired t-test was used to compare day 29 tumor volumes. Log-rank test was used for survival analysis. Chi-squared test was used to examine the correlation between treatment groups and metastasis rates. *, *p* < 0.05; **, *p* < 0.01; ***, *p* < 0.001

To assess the impact of combination anti-IL-1β and anti-PD-1 treatment on primary tumor growth, we utilized a previously established KPC orthotopic murine model of PDAC [33, 36]. The KPC-3403 cell line was derived from the primary tumor of a KPC mouse with lung metastasis only [34]. KPC-3403 tumors were inoculated into mice on Day 0 and mice were subsequently sorted into four treatment groups: (1) isotype control (human IgG, mouse IgG1), (2) anti-IL-1β, (3) anti-PD-1 and (4) anti-IL-1β + anti-PD-1 (*n* = 5 per group). Pre-treatment ultrasound of tumor-bearing mice was performed on day 4. Treatment for all groups started on Day 7 and was administered intraperitoneally for a total of three weeks: anti-IL-1β or the isotype control was administered every other day, and anti-PD-1 or the respective isotype control was administered twice a week (Fig. 1A). Tumor volume measurements were collected through ultrasound imaging once a week. After 23 days, we observed that the combination of anti-IL-1β and anti-PD-1 showed no significant difference in tumor size when compared to control, anti-IL-1β monotherapy, or anti-PD-1 monotherapy (Fig. 1B); however, combination therapy prolonged survival (Fig. 1C). These findings suggest the combination treatment of anti-IL-1β and anti-PD-1 has minimal effect on primary tumor growth. We thus hypothesize that the treatment prolongs the survival by suppressing lung metastasis. We would not be able to test this hypothesis in this experiment because mice died at the different times and were all found to have lung metastases. We thus tested the hypothesis in a more dedicate experiment below (Fig. 1D and E).

### Combination anti-IL-1β and anti-PD-1 slows primary tumor growth and prolongs survival in a PDAC murine orthotopic model with liver metastasis tropism

**Fig. 2:**
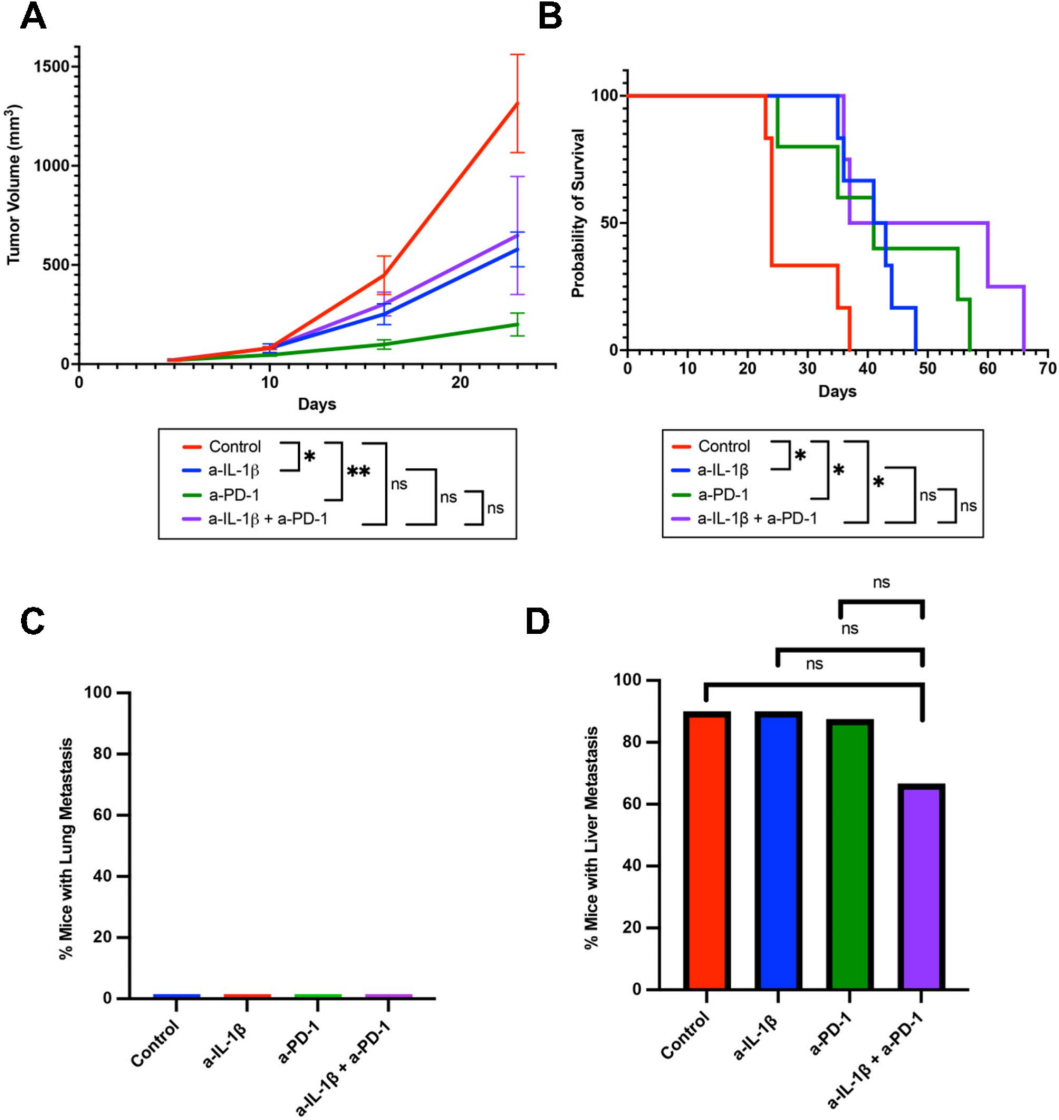
The combination of anti-IL-1β and anti-PD-1 prolonged survival but did not slow tumor growth or reduce liver metastasis rate in orthotopic PDAC mouse model with liver metastasis. (**A**) PDAC orthotopic tumor size was evaluated by US weekly until day 29 in mice treated with different combinations of anti-IL-1β and anti-PD-1 (*n* = 5 mice per group). (**B**) Kaplan-Meier survival curves of PDAC orthotopic model mice treated with different combinations of anti-IL-1β and anti-PD-1 (*n* = 5 mice per group). (**C**&**D**) When PDAC orthotopic tumor mice reached survival endpoint, necropsies were performed, and the number of mice with lung metastases (**C**) and liver metastases (**D**) for the KPC-4545 liver metastasis potential model were identified grossly and histologically (*n* = 8–10 mice per group). US, ultrasound. Unpaired t-test was used to compare day 29 tumor volumes. Log-rank test was used for survival analysis. Chi-squared test was used to examine the correlation between treatment groups and metastasis rates. *, *p* < 0.05; **, *p* < 0.01; ***, *p* < 0.001, by unpaired two-sided Students’ *t*-test

To study liver metastasis preclinically, our lab previously established a KPC-4545 cell line, which derived form a KPC mouse with liver metastasis only [34]. Using similar tumor inoculation methods and treatment strategies shown in Fig. 1A, we also examined the impact of the combination of anti-IL-1β and anti-PD-1 treatments on KPC-4545 orthotopic tumors. On day 23 after tumor implantation, we observed anti-IL-1β + anti-PD-1 had decreased tumor sizes compared to control (Fig. 2A). While anti-IL-1β monotherapy and anti-PD-1 monotherapy also reduced tumor volume compared to control, it was not to the magnitude of the anti-PD-1 + anti-IL-1β combination (Fig. 2A). Furthermore, the combination treatment significantly prolonged survival compared to control (Fig. 2B). These data suggested that the KPC-4545 orthotopic model may be sensitive to anti-IL-1β and anti-PD-1 combination treatment as evidenced by the decreased primary tumor growth and subsequently prolonged survival. Similarly, whether the treatments have an impact on liver metastases in this model was investigated in a dedicated experiment below (Fig. 2C and D).

### Combination anti-IL-1β and anti-PD-1 reduces lung metastasis rate in PDAC orthotopic model with lung metastasis tropism, but not liver metastasis rate in the model with liver metastasis tropism

Using a similar methodology described in Fig. 1A, we next sought to assess the effects of anti-IL-1β and anti-PD-1 treatment on the lung and liver metastasis rates in our PDAC orthotopic murine models with lung and liver metastasis tropism, respectively. Mice were again sorted in the following four treatment groups: (1) isotype control (human IgG, mouse IgG1), (2) anti-IL-1β, (3) anti-PD-1 and (4) anti-IL-1β + anti-PD-1. To this end, we implanted much larger tumors at 3–5 mm diameter, which resulted in approximately the same survival times among all the treatment groups. At necropsy, primary tumor sizes had no difference among all the treatment groups (Figure S1A; Figure S1B). We inspected all dissected lungs and livers for metastatic sites grossly and also histologically by H&E stain (Figure S1C; Figure S1D). We observed that the anti-IL-1β + anti-PD-1 combination treatment had decreased lung metastasis rates when compared to control, anti-IL-1β monotherapy and anti-PD-1 monotherapy in the KPC-3403 model with lung metastasis tropism (Fig. 1D). As expected, only one out of nine mice with the KPC-3403 orthotopic tumors developed liver metastasis (Fig. 1E). Similarly, for the KPC-4545 model with liver metastasis tropism, no mice developed lung metastasis as expected (Fig. 2C). However, neither monotherapies nor combination treatment significantly reduced the rate of liver metastasis (Fig. 2D). These data suggested that anti-IL-1β and anti-PD-1 combination treatment suppresses lung metastasis but has no effect on liver metastasis.

### The combination of anti-IL-1β and anti-PD-1 restores T cell activation negated by the monotherapies

To further investigate how anti-IL-1β and anti-PD-1 treatment modulates the tumor microenvironment, we performed flow cytometry analysis of tumor infiltrating leukocytes (TILs) from primary orthotopic tumors for both the KPC-4545 and the KPC-3403 model. Mice were sorted into four different treatment groups: (1) isotype control (human IgG, mouse IgG1), (2) anti-IL-1β, (3) anti-PD-1 and (4) anti-IL-1β + anti-PD-1. Mice were treated as described in Fig. 1A, but all tumors were harvested and TILs isolated for flow cytometry on Day 13 to determine the effects of treatment on T cells and their subpopulations (Figure S2). No difference in baseline was observed for either KPC-4545 or KPC-3403 in the percentages of CD4 + or CD8 + T cells among CD45 + cells or normalized cell count by 1 × 10^6 (Figure S3A; Figure S3B). Though anti-PD-1 monotherapy increased the percentage of CD45 + CD4 + cells in the KPC-4545 model, this effect was not observed in the KPC-3403 lung metastasis model. Combination treatment with anti-IL-1β and anti-PD-1 increased the percentage of the CD8 + T cells compared to following anti-PD-1 monotherapy in the KPC-3403 model only (Figure S3A).

**Fig. 3:**
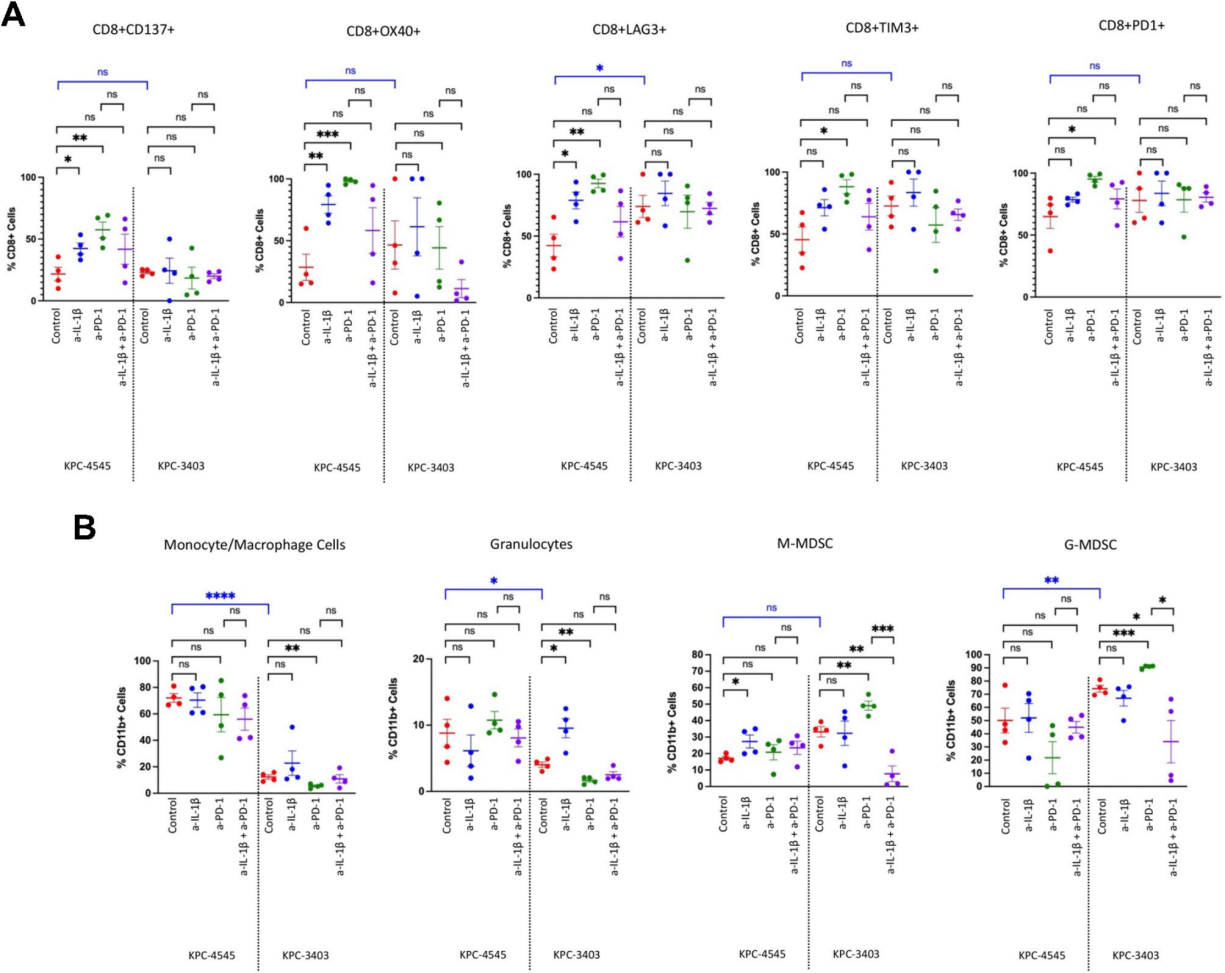
Anti-IL-1β and anti-PD-1 antibodies modulate T cell activity in the KPC-4545 liver metastasis potential model and myeloid cell composition in the KPC-3403 lung metastasis potential model. (**A**&**B**) Flow cytometry was performed on tumor infiltrating leukocytes from resected orthotopic tumor on day 13. The following tumor infiltrating leukocytes were analyzed: (**A**) percentage of CD137+, OX40+, LAG3+, TIM3+, and PD1 + cells among CD45 + CD8 + cells (*n* = 4 mice per group). (**B**) percentage of monocyte/macrophage cells, granulocytes, M-MDSC cells, and G-MDSC cells among CD45 + CD11b + cells (*n* = 4 mice per group) Data represent mean ± SEM. *, *p* < 0.05; **, *p* < 0.01; ***, *p* < 0.001, by unpaired two-sided Students’ *t*-test

Both anti-IL-1β and anti-PD-1 monotherapy increased the percentage of activated CD8 + CD137+, CD8 + OX40 + T cells and exhausted CD8 + LAG3+, CD8 + TIM3+, and CD8 + PD1 + T cells in the KPC-4545 model, but not in the KPC-3403 model (Fig. 3A). These findings suggest that the KPC-4545 orthotopic tumors may be sensitive to both anti-IL-1β and anti-PD-1 monotherapy in comparison to the KPC-3403 orthotopic tumor, as represented by how both treatments slowed tumor growth curve in the KPC-4545 model, but not the KPC-3403 model (Figs. 1B and 2A).

In the CD4 + T cell population, both anti-IL-1β and anti-PD-1 monotherapies reduced the percentage of CD4 + CD137 + T cells in the KPC-3403 model but increased the percentage of CD4 + OX40 + T cells in the KPC-4545 model (Figure S3C). Similarly, both anti-IL-1β and anti-PD-1 monotherapies increased the percentage of exhausted CD4 + LAG3+, CD4 + TIM3+, CD4 + PD1 + T cells in the KPC-4545 model, but not the KPC-3403 model (Figure S3C). Notably, combination treatment reduced the fraction of CD4 + PD1 + T cells compared to the anti-PD-1 monotherapy in the KPC-4545 model. No baseline difference for regulatory T cells (Tregs; CD4 + Foxp3+) was observed between the two models (Figure S3D; Figure S4). Furthermore, other than a decrease in the fraction of Tregs following anti-PD-1 monotherapy in the KPC-3403 model, no significant changes were noted in the percentage or normalized count of Tregs after treatment (Figure S3D). Therefore, although both anti-IL-1β and anti-PD-1 treatments individually may drive T cell exhaustion in KPC-4545 orthotopic model and may reduce T cell activation in the KPC-3403 model, their combinations would reverse this process.

### The combination of anti-IL-1β and anti-PD-1 reverses an increased trend of immunosuppressive myeloid cells as a result of monotherapy in the PDAC model with lung metastasis tropism, but not that with liver metastasis tropism

To further investigate the effects of anti-IL-1β and anti-PD-1 on the myeloid lineage, we performed flow analysis of myeloid subpopulations with TILs as described previously (Figure S5). We observed a clear baseline difference in the percentage of monocyte/macrophage (CD45 + CD11b + F4/80+) amongst CD11b + cells between the KPC-4545 and KPC-3403 models (Fig. 3B). Similarly, across all treatment groups, KPC-4545 orthotopic tumors had higher percentage of monocyte/macrophage compared to KPC-3403 tumors. The KPC-4545 model also had a higher baseline percentage of granulocytes (CD45 + CD11b + Ly6G+) compared to the KPC-3403 model, but the percentages of monocytic-myeloid derived suppressor cells (M-MDSC, CD45 + CD11b + Ly6C^high^Ly6G-) were comparable (Fig. 3B).

No changes to both the monocyte/macrophage and granulocyte populations were observed after treatment in the KPC-4545 model (Fig. 3B). In contrast, we noted that in the KPC-3403 model, anti-IL-1β monotherapy resulted in a non-significant trend of increase in monocyte/macrophage and a significant trend of increase granulocytes, respectively. anti-PD-1 treatment reduced the percentage of both monocyte/macrophages and granulocytes in the KPC-3403 model. Furthermore, in the KPC-3403 and not KPC-4545 tumors, we noted that anti-PD-1 monotherapy increased the percentage of M-MDSC and G-MDSC but addition of anti-IL-1β in the combination treatment reduced the percentage of both populations compared to baseline and anti-PD-1 monotherapy.

### Single nuclear RNA-seq analysis of KPC tumors with liver and lung metastasis potential

**Fig. 4:**
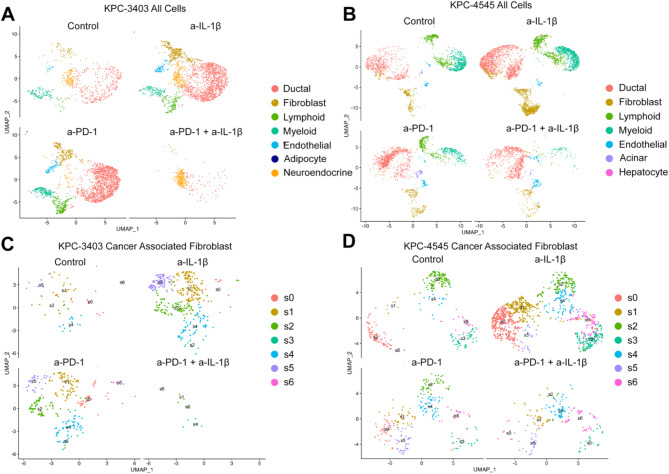
snRNA-seq of PDAC orthotopic tumor captures major cell types in the KPC-3403 lung metastasis model and the KPC-4545 liver metastasis model. (**A**&**B**) Uniform manifold approximation and projection (UMAP) embedding of single-nucleus profiles of representative cell types across the four treatment groups in the KPC-3403 model (**A**) and the KPC-4545 model (**B**) separated by treatment. (**C**&**D**) Re-clustered UMAP embeddings of CAFs across the four anti-IL-1β samples in the KPC-3403 (**C**) and the KPC-4545 model (**D**) stratified by treatment

We next examined the snRNA-seq data from both the KPC-3403 and KPC-4545 models to further compare the impact of anti-IL-1β treatment on the TME between the different models (Fig. 4). We noted that immune cells were less abundant in the KPC-3403 model compared to the KPC-4545 model in terms of both absolute cell count and fraction across the control, anti-IL-1β monotherapy, and anti-PD-1 monotherapy groups (Fig. 4A and B; Figure S6). It should be noted that the analysis was not feasible for the KPC-3403 tumor treated with the combination of anti-PD-1 and anti-IL-1β due to the small tumor size and thus low cell count in the snRNA-seq sample.

Our prior studies have demonstrated the differences in the CAF heterogeneity between KPC-3403 and KPC-4545 models underlying the organ-specific metastasis [34, 35]. Particularly, KPC-4545 tumors appear to have attenuated CAF heterogeneity whereas KPC-3403 tumors maintain the CAF heterogeneity in response to transforming growth factor TGFβ depletion [35]. As such, we examined the phenotype of the distinct CAF subpopulations in the four treatment groups relevant to this study (Fig. 4C and D). As noted previously, all clusters in the KPC-4545 model exhibited both myCAF and iCAF features at baseline (Figures S7A-C). Contrary to treatment with TGFβ-TRAP, anti-IL-1β treatment did not significantly alter CAF phenotype in the KPC-4545 model (Figure S7). Treatment with anti-IL-1β only made CAF Cluster 1 skew more clearly towards an iCAF phenotype (Figure S7D). However, while the CAF subpopulations in the KPC-3403 model demonstrated more distinct phenotypes, anti-IL-1β treatment similarly did not significantly alter the phenotypes of any of the three CAFs clusters in the KPC-3403 model (Figure S8). What appears to be more unique for the KPC-3403 model compared to the KPC-4545 model is the smaller number of CAFs in the untreated control tumor and the increase of CAFs following anti-IL-1β treatment (Fig. 4). Again, it should be noted that CAFs in the KPC-3403 tumor following the anti-IL-1β + anti-PD-1 combo treatment are not assessable due to the small tumor size.

We next examined the lymphoid cell populations. While we were able to identify major clusters, including CD8 + T cells, CD4 + T cells, Tregs, B cells, and plasma cells in the lymphoid population and granulocytes and macrophages in the myeloid population, a more in-depth analysis of immune cells is limited due to the reduced number of immune cells in the KPC-3403 tumor (Figure S9).

### Differential gene expression analysis of multiple cell types following anti-IL-1β treatment in the KPC tumors with liver and lung metastasis potential respectively

**Fig. 5:**
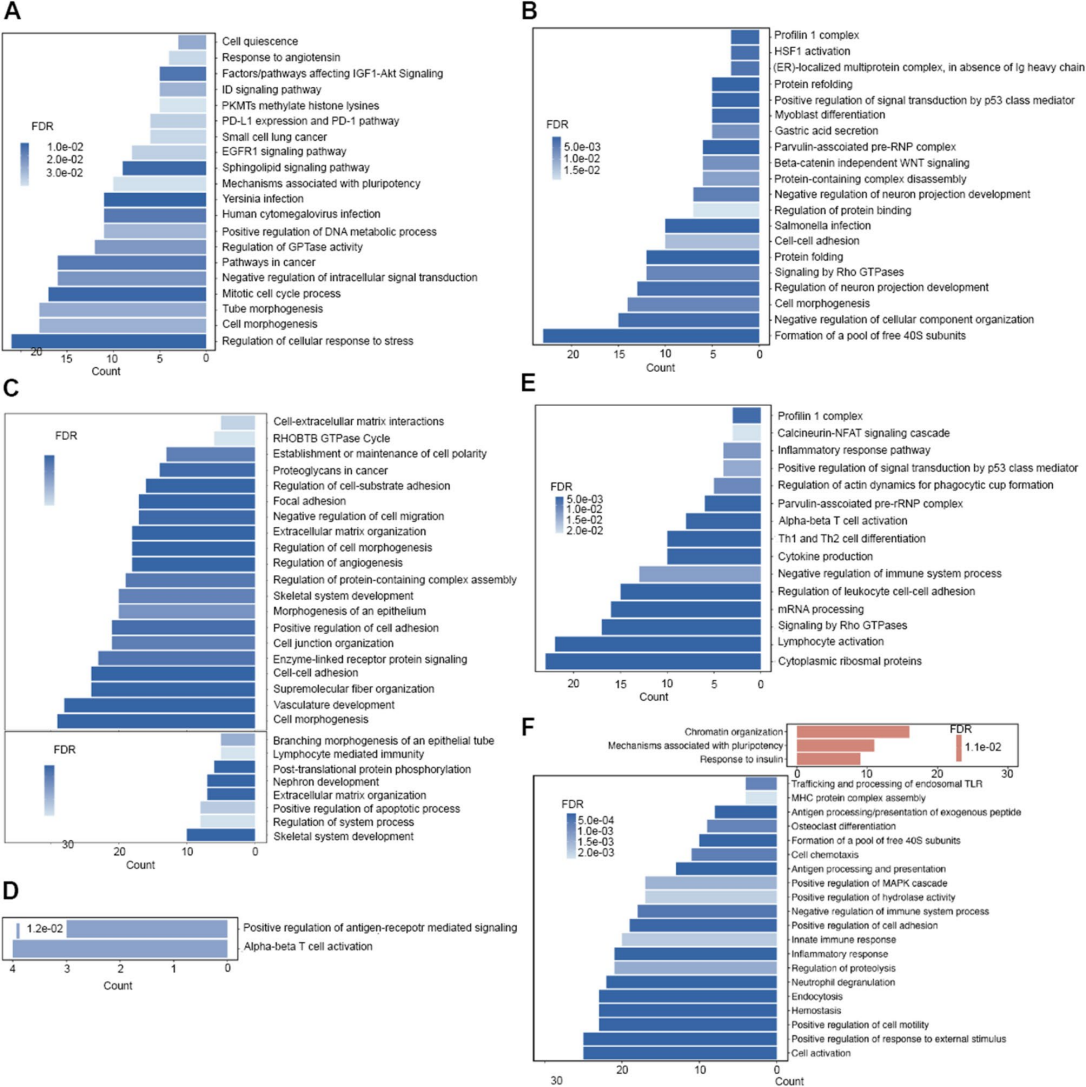
Pathway analysis of differentially expressed genes after anti-PD-1 and anti-IL-1β treatment. (**A**-**B**) Top significant pathways (FDR < 0.05) from significant differentially expressed genes (Log_2_FC<-0.5 or Log_2_FC > 0.5 and adjusted p-value < 0.05) between anti-PD-1- and anti-PD-1 + anti-IL-1β combination-treated samples for ductal cells in the KPC-3403 (**A**) and KPC-4545 model (**B**). (**C**-**F**) Top significant pathways between control and anti-IL-1β-treated samples of the KPC-4545 model in CAF clusters 1 and 2 (**C**), CD8 T cells (**D**), CD4 T cells (**E**), and macrophages (**F**). Blue and red indicate downregulated and upregulated after additional treatment, respectively. FDR, false discovery rate; FC, fold change

We then conducted differential gene expression analysis on cell types of interest, including ductal, CAFs, and immune cells, between the control treatment group and anti-IL-1β treatment group or the anti-PD-1 versus combination treatment group, followed by the pathway analysis (Fig. 5). While multiple significantly enriched pathways from differentially expressed genes (DEGs) in the anti-PD-1 versus combination treated samples overlapped between the KPC-3403 and KPC-4545 models, the negative regulation of neuron projection development pathway was unique for the ductal cells in the KPC-4545 tumor (Fig. 5A and B).

Given anti-IL-1β treatment resulted in an increase in all CAF subpopulations in the KPC-3403 tumor, no obvious DGE was observed in the control versus anti-IL-1β treated samples as anticipated. In contrast, enriched pathways downregulated by anti-IL-1β treatment in the KPC-4545 CAF clusters included various pathways in cytoskeleton rearrangement, morphogenesis, adhesion and migration (Fig. 5C). Thus, anti-IL-1β treatment appears to suppress the function of CAFs in the KPC-4545 tumors but does not alter the function of CAFs in the KPC-3403 tumors.

Similarly, anti-IL-1β treatment increased the infiltration of CD8 + and CD4 + T cells in the KPC-3403 tumor compared to the control sample; and no significant DEGs were identified in the control versus anti-IL-1β treated samples. On the other hand, in the KPC-4545 CD8 + T cell population, pathways enriched in the DEGs downregulated by anti-IL-1β treatment included ɑβ T cell activation (Fig. 5D). Again, the pathways enriched in the DEGs downregulated by anti-IL-1β treatment in the CD4 + T cells included lymphocyte activation and ɑβ T cell activation (Fig. 5E). Thus, anti-IL-1β treatment appears to suppress the activation of T cells in the KPC-4545 tumors but does not appear to alter the function of T cells in the KPC-3403 tumors.

Furthermore, while we did not identify DEGs after anti-IL-1β treatment in the KPC-3403 macrophage population, we found that anti-IL-1β treatment led to the downregulation in the pathways such as antigen presentation, innate immune and inflammatory response, and MHC protein complex assembly in the macrophage population in the KPC-4545 tumors (Fig. 5F). This finding suggests that anti-IL-1β treatment suppresses the innate immune response in macrophages in the KPC-4545 tumors but does not appear to alter the function of macrophages in the KPC-3403 tumors.

Taken together, while anti-IL-1β treatment appears to enhance the CAF and immune cell infiltration in the KPC tumor with lung metastasis potential, it appears to suppress the function of CAF and immune cells in the KPC tumor with liver metastasis potential.

### Anti-IL-1β antibody modulates the cytokine/chemokine ligand-receptor interactions in KPC tumors with different organ-specific metastasis distinctly

**Fig. 6:**
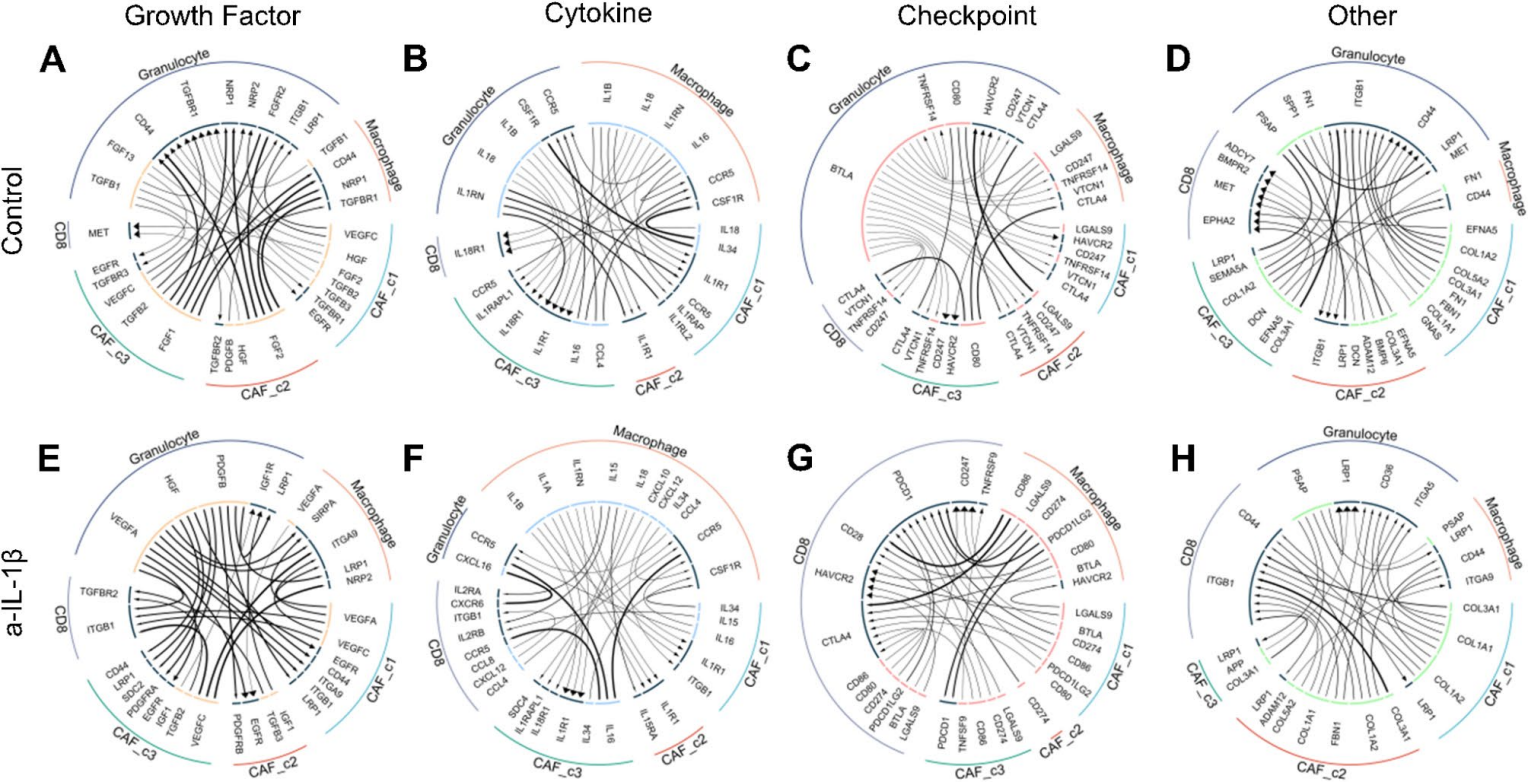
Ligand receptor analysis reveals changes in interactions between CAFs and immune cells after anti-IL-1β and anti-PD-1 treatment in the KPC-3403 model. Chord diagram of ligand-receptor interactions between all three major clusters of CAFs, CD8 T cells, macrophages, and granulocytes. Each row represents a treatment sample in the order of control (**A**-**D**) and anti-IL-1β treated (E-H). Each column represents a category defined by iTALK, including growth factor, cytokines, checkpoint, and other going from left to right

To understand the interactions between different cell types and the impact of anti-IL-1β treatment on such interactions, we used the iTALK package to infer ligand-receptor interaction with the snRNA-seq data. Here we focused on four cell types including, CAFs, CD8 + T cells, macrophages, and granulocytes. Interactions were categorized into growth factor, cytokines, checkpoint, and other based on the type of ligands. We compared the top thirty ligand-receptor interactions within each category among different treatment groups (Fig. 6; Figure S10; Tables S1-S5).

Similar to our previous finding for the KPC-4545 model [35], all three clusters of CAFs appeared to be the main source of growth factors, including TGFβs, FGFs, and VEGFs which bind to receptors including CD44, TGFβreceptors, and ITGs in the untreated KPC-3403 tumor (Fig. 6A; Figure S10A). Such a pattern did not appear to have changed following anti-IL-1β or anti-PD-1 treatment (Tables S1-S4). However, following anti-IL-1β treatment, SIRPa arose as the receptor on macrophages for VEGFs in the KPC-4545 tumor (Table S1). Nevertheless, in the KPC-3403 tumor (Table S2), FGFs did not appear to be one of dominant ligands anymore. In contrast, VEGF-A became a main ligand in replacement of VEGF-C, and ITGs became one of main receptors for both VEGF-A and -C.

As previously shown in the untreated KPC-4545 tumor (Tables S1-S4), IL-1 signaling, including IL-1ɑ, IL-1β, and IL-18, also appeared to be the dominant cytokine-type ligand-receptor interaction between macrophages/granulocytes and CAFs for the KPC-3403 model (Tables S1&S2). However, in the untreated KPC-4545 (Table S1), CXCL10, CCL13 and CCL8 were also presented as the cytokine ligands. Following anti-IL-1B treatment, IL-15 became a more dominant ligand in both KPC-4545 and KPC-3403 tumors. Nevertheless, while CCL5 became a more dominant ligand for CCR5 in the KPC-4545 tumor (Table S1), CCL4 remained a dominant ligand for CCR5 and CXCL12, CXCL10, and CXCL16 arose as major ligands for different receptors in the KPC-3403 model following anti-IL-1β treatment (Table S2).

No significant changes in the co-stimulatory and checkpoint interactions were observed in the KPC-4545 model following anti-IL-1β treatment except for the disappearance of CD40LDG as a ligand (Figure S10C; Figure S10G; Table S1). On the other hand, though very few co-stimulatory and checkpoint interactions were present in the KPC-3403 tumor at baseline (Fig. 6C; Table S2), these interactions were restored to a similar pattern compared to that of the KPC-4545 model following anti-IL-1β treatment. Together, the iTALK analysis suggests that anti-IL-1β treatment modulates the ligand-receptor signaling differently in KPC tumors with distinct organ-specific metastasis, particularly the FGF, VEGF, CCL5, IL-15, CXC10, CCL13, and CCL18 ligands and the co-stimulatory and checkpoint signaling.

On the other hand, anti-PD-1 treatment did not modulate ligand-receptor interactions in the KPC-3403 tumor significantly (Table S3). This was the same for the KPC-4545 model except for checkpoint signaling (Table S4). Nevertheless, the combination of anti-PD-1 and anti-IL-1β treatments (Table S5) resulted in similar modulations of the cytokine/chemokine ligand-receptor interactions as compared to anti-IL-1β treatment (Table S1) in the KPC-4545 tumor.

## Discussion

This study represents the first attempt of investigating the antitumor immune response of anti-IL-1β and anti-PD-1 combination therapy in the context of CAF heterogeneity and one of the first few attempts of addressing the efficacy of immunotherapy broadly in such a context. More specifically, this study found that the combination of anti-IL-1β and anti-PD-1 treatment does not slow primary tumor growth but reduces lung metastasis rate and prolongs overall survival in PDAC orthotopic murine model with lung metastasis tropism. In contrast, combination anti-IL-1β and anti-PD-1 slows primary tumor growth in a PDAC murine orthotopic model with liver metastasis tropism, but does not reduce liver metastasis rate. Although these mouse models may not fully resemble human PDAC with liver or lung metastasis tropism, they provide an avenue for studying antitumor immune response in the context of TME and CAF heterogeneity.

Despite being limited by low cell numbers, snRNA-seq analysis showed that anti-IL-1β treatment does not alter CAF heterogeneity in both tumors with liver or lung metastasis tropism, respectively. However, anti-IL-1β treatment appears to enhance CAF and immune cell infiltration without altering their functions in the KPC tumor with lung metastasis tropism. On the other hand, it may suppress the function of CAF and immune cells in the KPC tumor with liver metastasis tropism. Flow cytometry of tumor infiltrating immune cells also showed an increased trend of immunosuppressive myeloid cells and exhausted T cells as a result of anti-IL-1β monotherapy, but the combination of anti-IL-1β and anti-PD-1 reverses these changes in the PDAC model with lung metastasis tropism, but not in the model with liver metastasis tropism. Therefore, the distinct response in CAFs and immune cells could explain the differential treatment response in the lung metastasis versus liver metastasis.

This study is limited by having not compared CAF and immune cell response between primary tumor and metastasis, which shall be further investigated in future studies. In addition, only one murine model is available for liver metastasis tropism and lung metastasis tropism, respectively. Moving forward, more murine models for liver metastasis and lung metastasis tropisms would need to be established and used to validate the findings in this study.

Although this study supported the hypothesis that anti-tumor immune response of the combination of anti-IL-1β antibody and anti-PD-1 antibody is context-dependent by showing the antitumor immune response is affected by the stromal heterogeneity in PDACs, it is limited by its hypothesis-generating nature in most parts of the study. Future studies will further test the hypothesis that the observed CAF and immune response in the TME is the underlying mechanism of distinct responses to the combination of anti-IL-1β and anti-PD-1 in the primary tumor versus metastasis and different changes in the metastasis rate to the liver versus to the lung.

## Electronic supplementary material

Below is the link to the electronic supplementary material.

Supplementary Material 1

Supplementary Material 2: Supplementary Table 1 (Table.S1): Top 30 Ligand Receptor Interactions for Control versus anti-IL-1β Treated Samples in KPC-4545 Liver Metastasis Tropism for Comparison. Separated into four different ligand receptor interactions: growth factor, cytokines, checkpoint, and other

Supplementary Material 3: Supplementary Table 2 (Table.S2): Top 30 Ligand Receptor Interactions for Control versus anti-IL-1β Treated Samples in KPC-3403 Lung Metastasis Tropism for Comparison. Separated into four different ligand receptor interactions: growth factor, cytokines, checkpoint, and other

Supplementary Material 4: Supplementary Table 3 (Table.S3): Top 30 Ligand Receptor Interactions for Control versus anti-PD-1 Treated Samples in KPC-3403 Lung Metastasis Tropism for Comparison. Separated into four different ligand receptor interactions: growth factor, cytokines, checkpoint, and other

Supplementary Material 5: Supplementary Table 4 (Table.S4): Top 30 Ligand Receptor Interactions for Control versus anti-PD-1 Treated Samples in KPC-4545 Liver Metastasis Tropism for Comparison. Separated into four different ligand receptor interactions: growth factor, cytokines, checkpoint, and other

Supplementary Material 6: Supplementary Table 5 (Table.S5): Top 30 Ligand Receptor Interactions for anti-PD-1 versus Combination Treated Samples in KPC-4545 Liver Metastasis Tropism for Comparison. Separated into four different ligand receptor interactions: growth factor, cytokines, checkpoint, and other

## Data Availability

Mouse snRNA-seq data is available in the NCBI’s GEO database (GSE275596).
