# Supplementary material for: Distinct response to IL-1β blockade in liver- and lung-specific metastasis mouse models of pancreatic cancer with heterogeneous tumor microenvironments
Source: Exp Hematol Oncol. 2025 Feb 13;14:13. doi: 10.1186/s40164-025-00607-w (PMC11823153; doi:10.1186/s40164-025-00607-w)
Supplement: Supplementary file 1 — Supplementary Material 1 [file 40164_2025_607_MOESM1_ESM.pdf]

SUPPLEMENTAL FIGURE LEGENDS

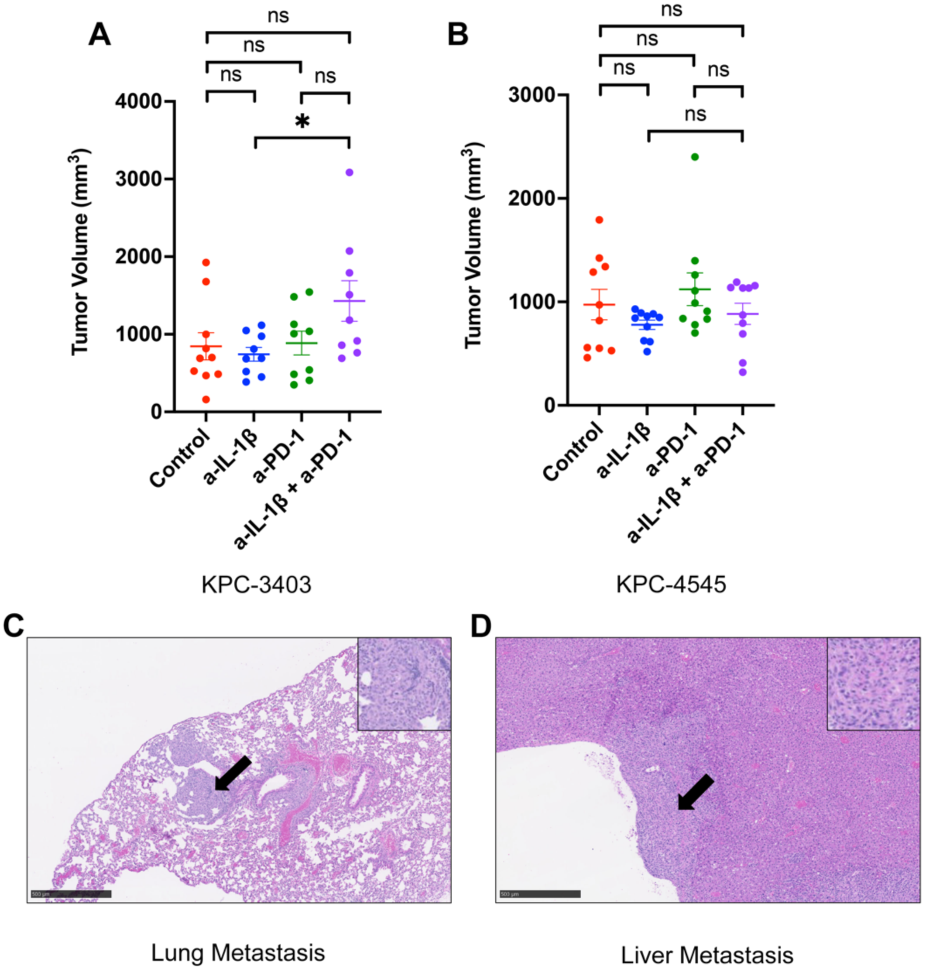

Figure S1. Tumor volume at necroscopy and representative H&E images of liver and lung metastatic sites in PDAC orthotopic mouse model syngeneic wild-type C57Bl/6 mice.

(A-B) Tumor volume at necroscopy across different treatments of anti-PD-1 and anti-IL-1β as measured by a caliper in the KPC-3403 (A) and KPC-4545 model (B). **(C-D)** 5X magnification of a lung metastasis site **(C)** and a liver metastasis site **(D)** and metastastic regions (arrow) are magnified and displayed in top right corner, respectively.

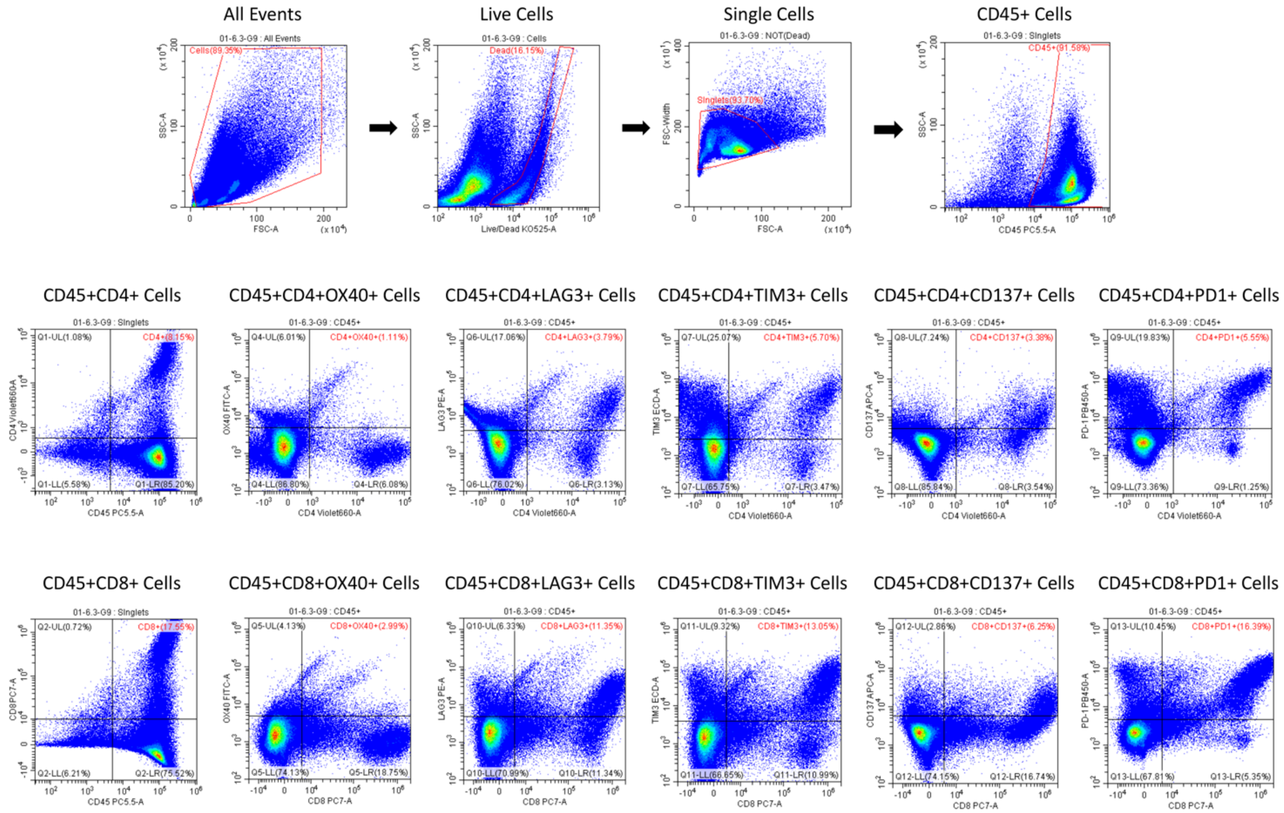

Figure S2. Flow cytometry gating strategy for identification of effector T cells.

Dead cells were excluded by gating on cells negative for viability marker Aqua Blue. Forward scatter width (FSC-W) and scatter area (FSC-A) were used to exclude doublets. CD45+ T-cells were identified. The flow cytometry gating strategy of different effector T-cell types are shown.

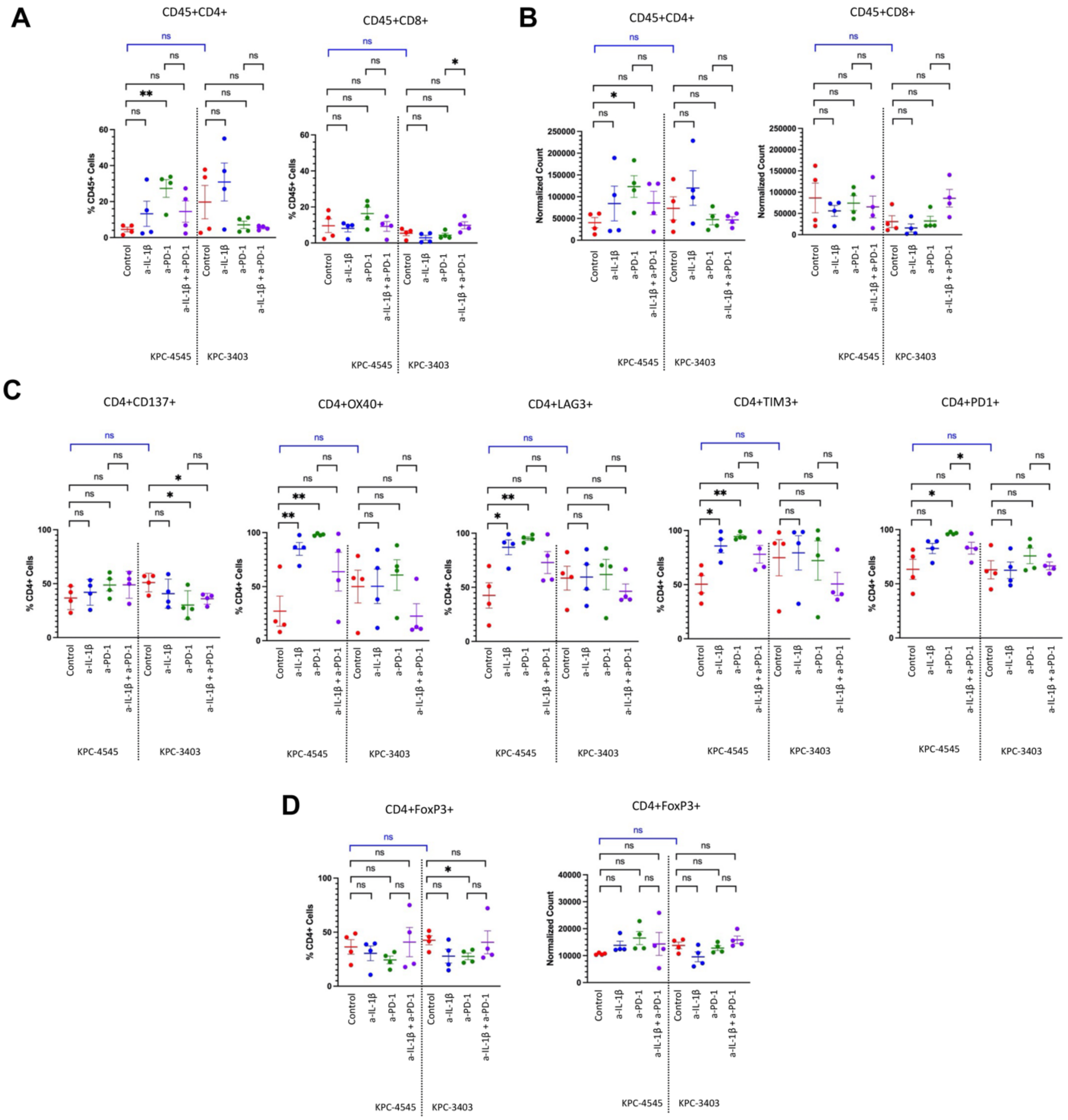

Figure S3. The combination of anti-PD-1 and anti-IL-1β treatment had comparable levels of CD45+CD8+ T cells, CD45+CD4+ T cells, and regulatory T cells but modulates the activation and exhaustion of tumor infiltrating CD4+ T cells in the KPC-3403 model.

(A-D) Flow cytometry was performed on isolated tumor infiltrating leukocytes from resected orthotopic tumor on day 13 The following isolated tumor-infiltrating leukocytes were analyzed

(n=4 mice per group): **(A)** percentage of CD4+ and CD8+ cells among CD45+ cells, **(B)** normalized cell counts of CD4+ and CD8+ cells per 1 x 106 cells, **(C)** percentage of CD137+, OX40+, LAG3+, TIM3+, and PD1+ cells among CD45+CD4+ cells, **(D)** percentage of FoxP3+ cells among CD4+ cells (n = 4 mice per group), normalized cell counts of CD4+FoxP3+ per 1 x 106 cells (n = 4 mice per group). *, $p<0.05$; **, $p<0.01$; ***, $p<0.001$, by unpaired t-test. Data represent mean ± SEM.

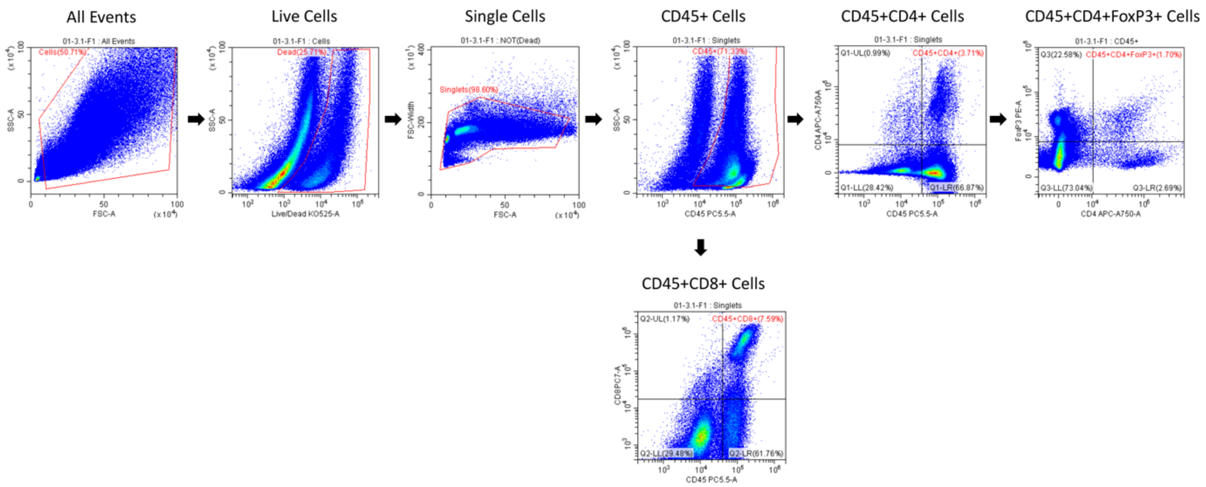

Figure S4. Flow cytometry gating strategy for identification of regulatory T-cells.

Dead cells were excluded by gating on cells negative for viability marker Aqua Blue. Forward scatter width (FSC-W) and scatter area (FSC-A) were used to exclude doublets. CD45+ cells were identified. Regulatory T-cells were defined as CD45+CD4+FoxP3+ cells.

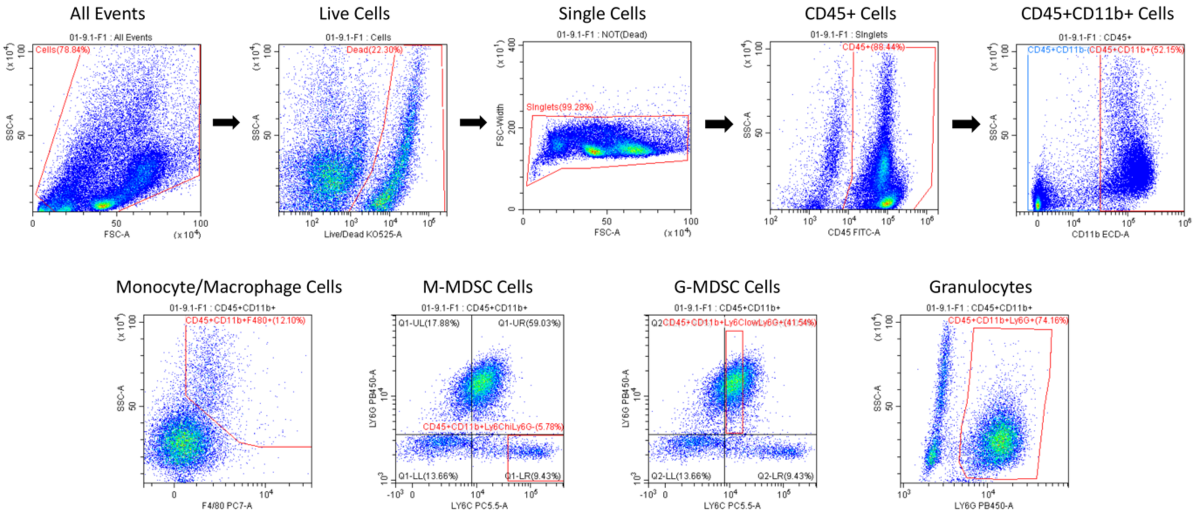

Figure S5. Flow cytometry gating strategy for identification of myeloid cell subtypes.

Dead cells were excluded by gating on cells negative for viability marker Aqua Blue. Forward scatter width (FSC-W) and scatter area (FSC-A) were used to exclude doublets. CD45+CD11b+ cells were identified. Monocyte/macrophage cells were defined as CD45+CD11b+F4/80+ cells. M-MDSC cells were defined as CD45+CD11b+Ly6C^{high}Ly6G- cells. G-GMDSC cells were defined as CD45+CD11b+Ly6C^{low}Ly6G+ cells. Granulocytes were defined as CD45+CD11b+Ly6G+ cells.

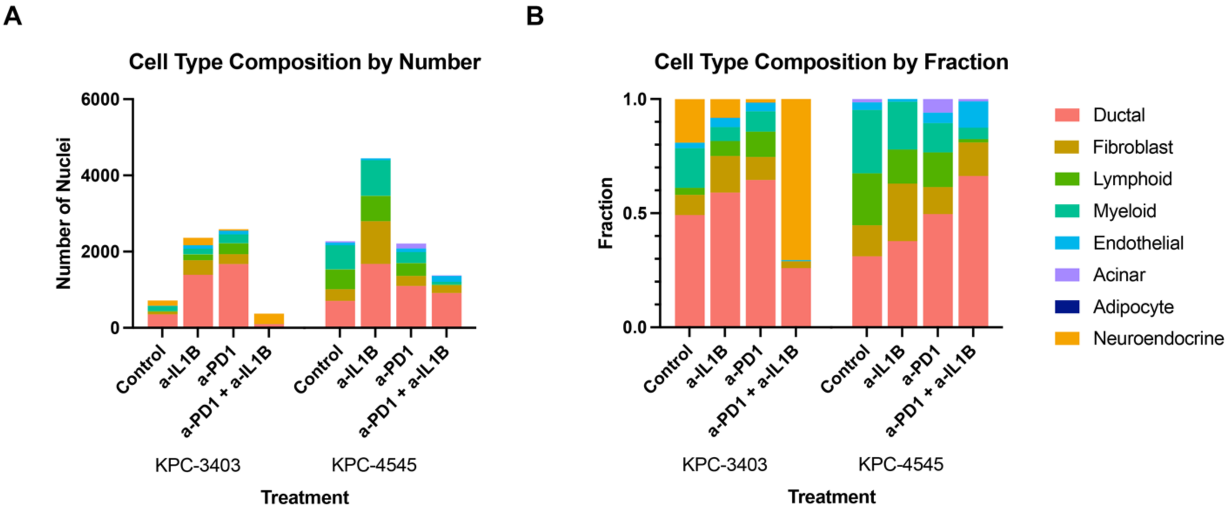

Figure S6. Cell Type Distribution in the KPC-4545 and KPC-3403 Model.

(A-B) Distribution of each cell type for each individual sample by cell count **(A)** and fraction of total nuclei **(B)** in each sample.

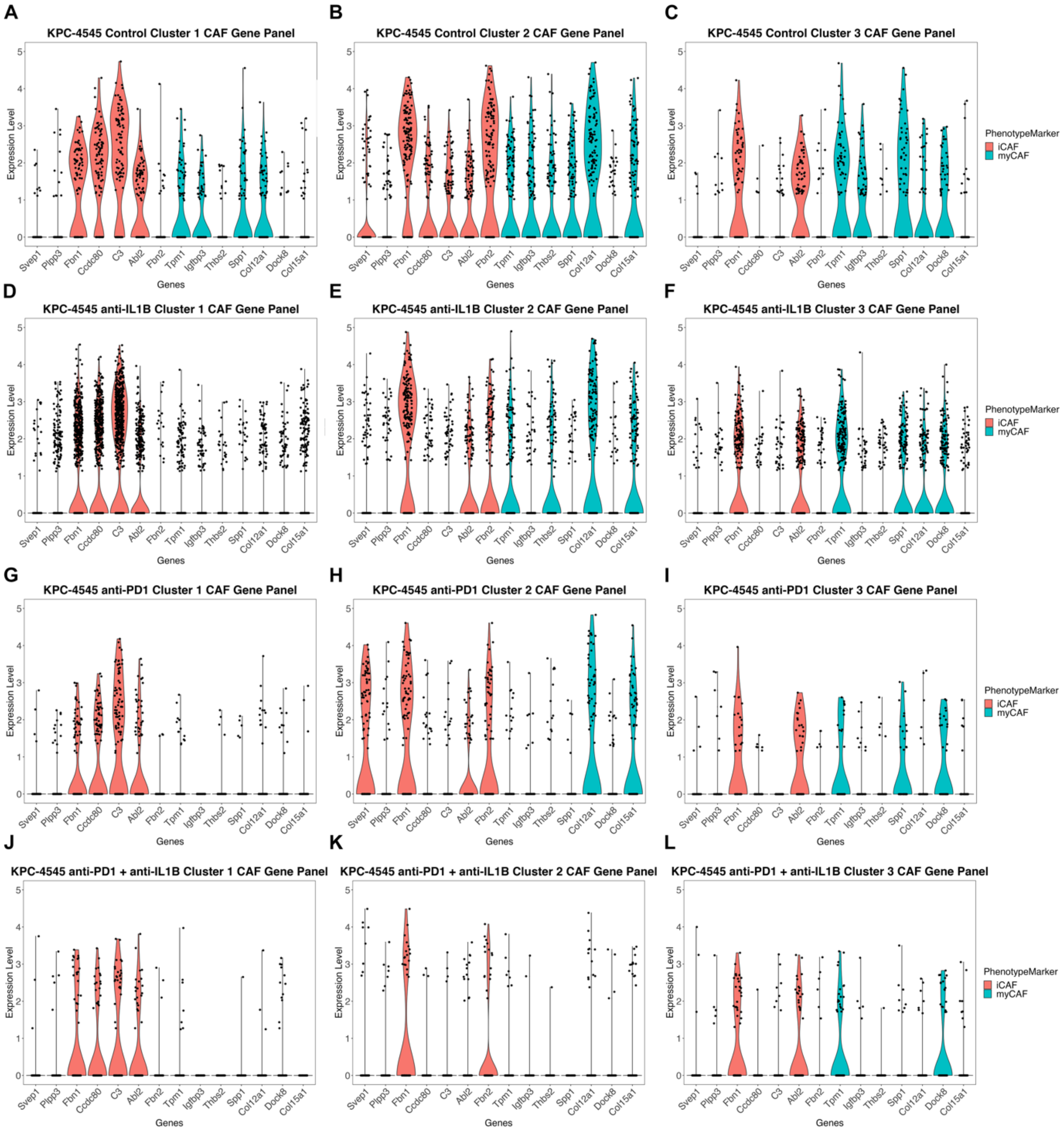

Figure S7. Anti-IL-1β antibody does not singificantly modulate CAF phenotype across different CAF clusters in the KPC-4545 model.

(A-L) CAF phenotype gene panel with iCAF (red) and myCAF (blue) signature genes across the three major KPC-4545 CAF clusters of the control **(A-C)**, a-IL-1β treated **(D-F)**, a-PD-1 treated **(G-I)**, and combination treated samples **(J-L)**.

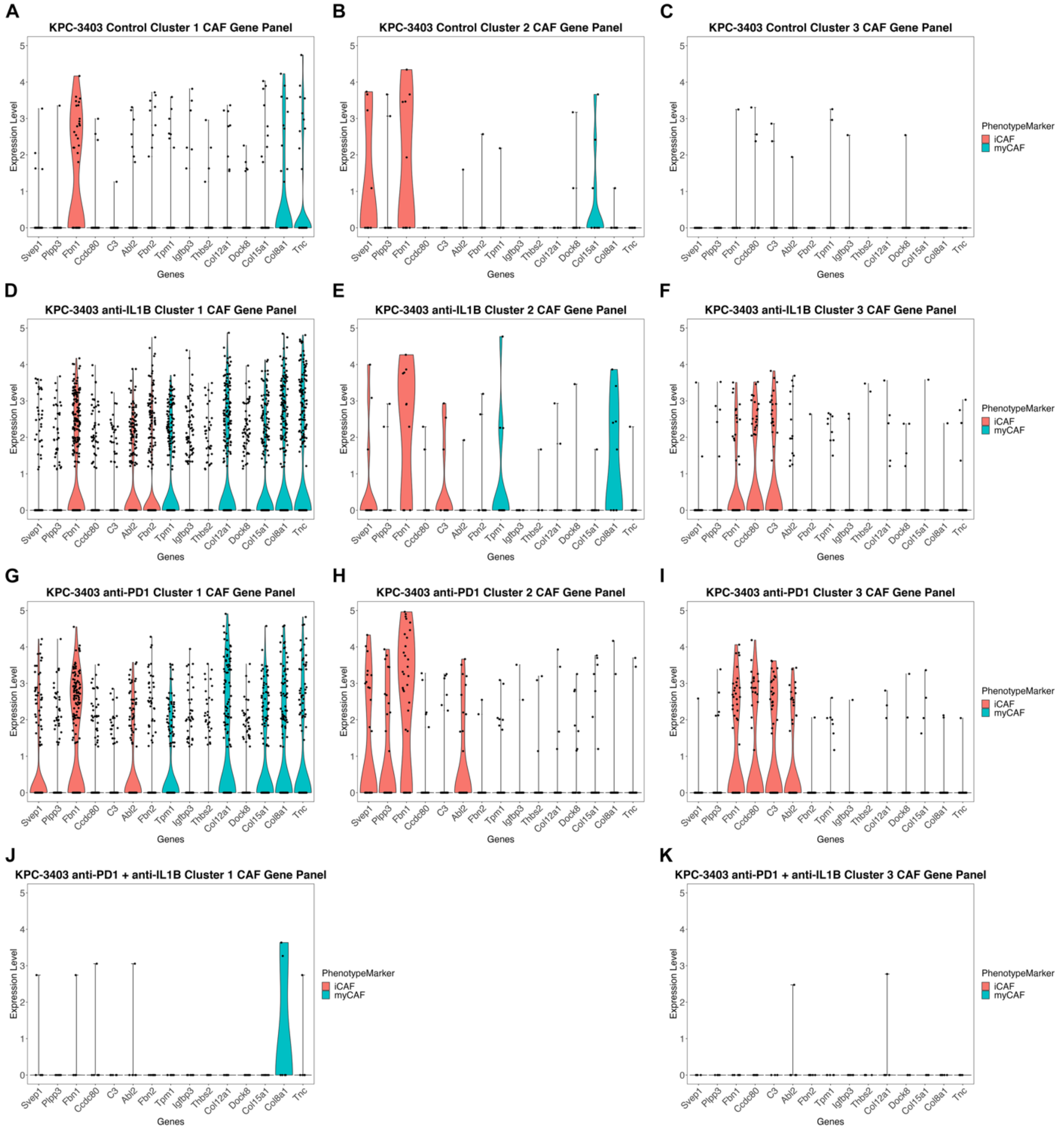

Figure S8. Anti-IL-1β antibody does not modulate CAF phenotype across different CAF clusters in the KPC-3403 model.

(A-K) CAF phenotype gene panel with iCAF (red) and myCAF (blue) signature genes across the three major KPC-3403 CAF clusters in the control **(A-C),** a-IL-1β**-**treated **(D-F)** a-PD-1-treated

(G-I), and combination-treated **(J-K)** samples. No cells were observed in cluster 2 for the combo treated sample.

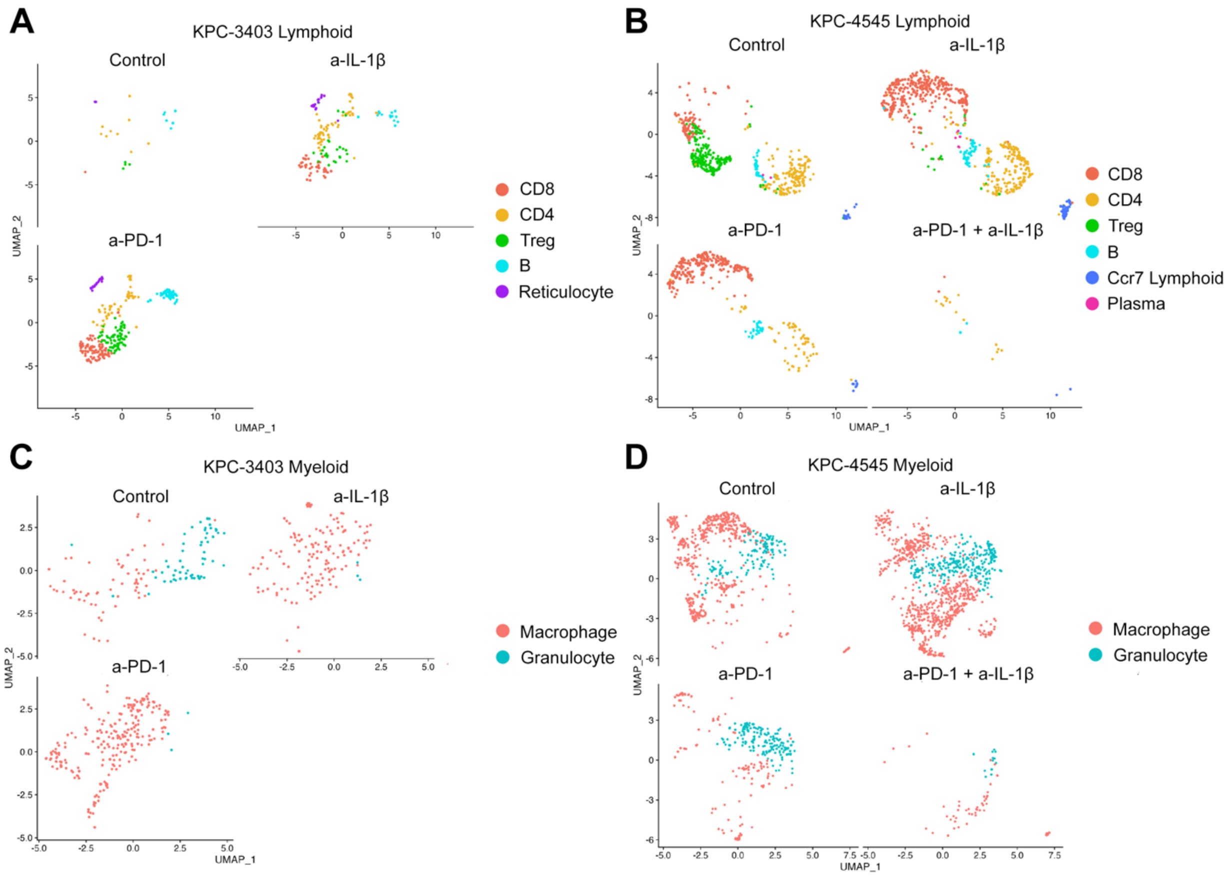

Figure S9. Lymphoid and myeloid landscape of the KPC-3403 and KPC-4545 tumor model following treatment with anti-PD-1 and anti-IL-1β.

(**A&B)** Re-clustered uniform manifold approximation and projection (UMAP) embeddings of annotated lymphoid cells across the four a-IL-1β samples in the KPC-3403 **(A)** and the KPC-4545 model **(B)** stratified by treatment. **(C&D)** Re-clustered UMAP embeddings of annotated myeloid cells across the four a-IL-1β samples in the KPC-3403 **(C)** and the KPC-4545 model **(D)** stratified by treatment.

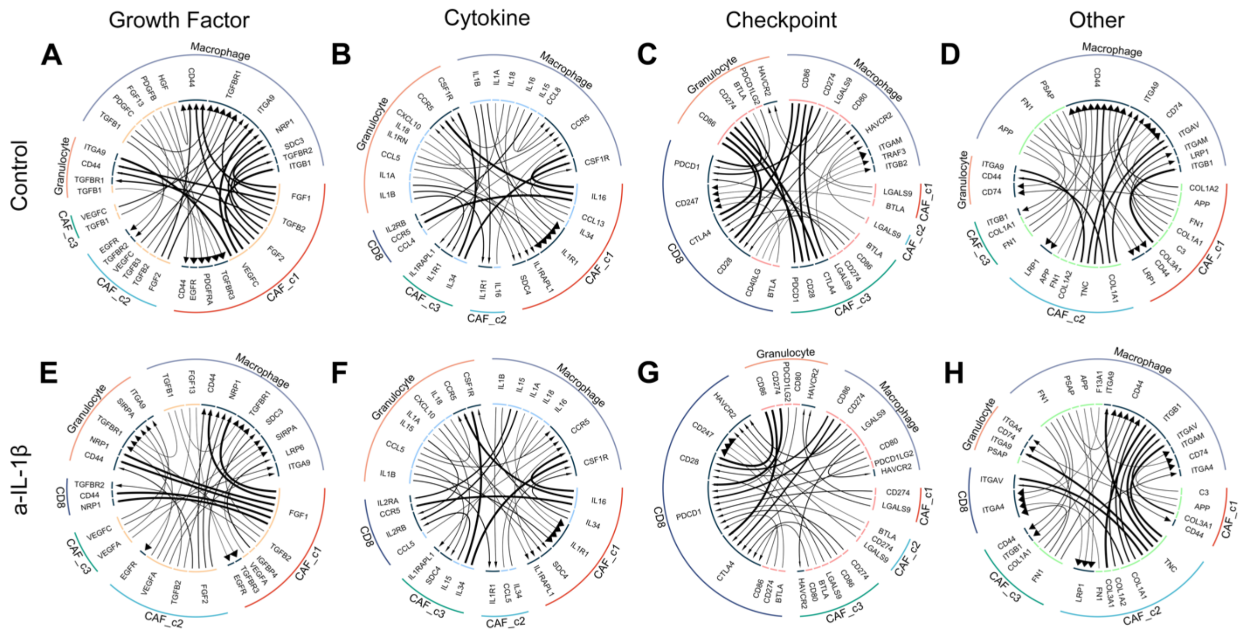

Figure S10. Ligand receptor analysis reveals complex ligand-receptor interactions between CAFs and immune cells in the KPC-4545 model.

Chord diagram of ligand-receptor interactions between all three major clusters of CAFs, CD8 T cells, macrophages, and granulocytes. Each row represents a treatment sample in the order of control **(A-D) and** a-IL-1β **(E-H)** from top to bottom. Each column represents a category defined by iTALK, including growth factor, cytokines, checkpoint, and other going from left to right.

SUPPLEMENTAL TABLE DESCRIPTIONS

Supplementary Table 1 (Table.S1): Top 30 Ligand Receptor Interactions for Control versus anti-IL-1β Treated Samples in KPC-4545 Liver Metastasis Tropism for Comparison. Separated into four different ligand receptor interactions: growth factor, cytokines, checkpoint, and other.

Supplementary Table 2 (Table.S2): Top 30 Ligand Receptor Interactions for Control versus anti-IL-1β Treated Samples in KPC-3403 Lung Metastasis Tropism for Comparison. Separated into four different ligand receptor interactions: growth factor, cytokines, checkpoint, and other.

Supplementary Table 3 (Table.S3): Top 30 Ligand Receptor Interactions for Control versus anti-PD-1 Treated Samples in KPC-3403 Lung Metastasis Tropism for Comparison. Separated into four different ligand receptor interactions: growth factor, cytokines, checkpoint, and other.

Supplementary Table 4 (Table.S4): Top 30 Ligand Receptor Interactions for Control versus anti-PD-1 Treated Samples in KPC-4545 Liver Metastasis Tropism for Comparison. Separated into four different ligand receptor interactions: growth factor, cytokines, checkpoint, and other.

Supplementary Table 5 (Table.S5): Top 30 Ligand Receptor Interactions for anti-PD-1 versus Combination Treated Samples in KPC-4545 Liver Metastasis Tropism for

Comparison. Separated into four different ligand receptor interactions: growth factor, cytokines, checkpoint, and other.
